# A Poly-(ethylene glycol)-diacrylate 3D-Printed Micro-Bioreactor for Direct Cell Biological Implant-Testing on the Developing Chicken Chorioallantois Membrane

**DOI:** 10.3390/mi13081230

**Published:** 2022-07-31

**Authors:** Eric Lutsch, Andreas Struber, Georg Auer, Thomas Fessmann, Günter Lepperdinger

**Affiliations:** System-Precision-on-Chip—SPOC Laboratories, Department of Biosciences and Medical Biology, University Salzburg, Hellbrunnerstrasse 34, A-5020 Salzburg, Austria; eric.lutsch@plus.ac.at (E.L.); andreas.struber@plus.ac.at (A.S.); georg.auer@gmail.com (G.A.); thomas.fessmann@stud.sbg.ac.at (T.F.)

**Keywords:** in vivo micro bioreactor, additive manufacturing, poly-(ethylene glycol)-diacrylate, biocompatibility

## Abstract

Advancements in biomaterial manufacturing technologies calls for improved standards of fabrication and testing. Currently 3D-printable resins are being formulated which exhibit the potential to rapidly prototype biocompatible devices. For validation purposes, 3D-printed materials were subjected to a hierarchical validation onto the chorioallantoic membrane of the developing chicken, better known as the HET CAM assay. Working along these lines, prints made from poly-(ethylene glycol)-diacrylate (PEGDA), which had undergone appropriate post-print processing, outperformed other commercial resins. This material passed all tests without displaying adverse effects, as experienced with other resin types. Based on this finding, the micro bioreactors (MBR) design, first made of PDMS and that also passed with cell tests on the HET-CAM, was finally printed in PEGDA, and applied in vivo. Following this workflow shows the applicability of 3D-printable resins for biomedical device manufacturing, consents to adherence to the present standards of the 3R criteria in material research and development, and provides flexibility and fast iteration of design and test cycles for MBR adaptation and optimization.

## 1. Introduction

Technologies used in medical biology are constantly evolving through incorporation of new techniques, e.g., 3D-printing [1]. Additive manufacturing (AM) provides a novel means for device production not only in engineering but also for biomedical applications. This manufacturing technology eases fabrication, which, based on traditional methods such as machining or molding, would otherwise be very challenging. In line with such desideration and prior to applications as complex as for clinical use, processed material and composites need to be carefully selected. This may eventually also include animal testing. By now, contemporary AM procedures have been developed, which allow almost everybody to print any type of object. To guide translation of this enabling technology, valid standards for material processing and eventually testing must be established. 

Three-dimensional-printing approaches that support and enable a plethora of biomedical applications have previously been described [2,3]. To date, this technology is increasingly employed for the prototyping of devices that are used for characterization of living cells in culture. Eventually, the printing of pieces of implantable devices by means of AM is also greatly desired.

For successful implementation of 3D-printed objects, an appropriate evaluation of 3D-printed polymers after photo-polymerization is both critical and pivotal for understanding the physicochemical properties of a biomaterial, especially in the context of effectuating cellular physiology. Bioassays need to be applied for sensitive evaluation of chemicals that potentially leach out from 3D-prints. The manufactured materials are submersed in cell culture media for prolonged periods and the conditioned media including leachables are applied on 2D cell cultures [4]. Hence, this type of assay may also inform about cytocompatibility in the case when cells are in close contact or even adhere to biomaterials. Despite this important first reference point, it will however not yield full information regarding the material’s biocompatibility. Recent studies investigating the biocompatibility of 3D-printable resins most commonly assess cytocompatibility regarding cytotoxic assays [4,5]. As the field lacks specifications for labels and declarations regarding cyto-/biocompatibility, printable material is currently synthesized, selected, and tested for applications in a biological context [6,7,8].

As a first conducive step towards reaching these goals, we here addressed questions not only for rapid and inexpensive manufacturing of devices suitable for research purposes in cell and tissue culture, but also in combination with organ-on-a-chip technology, a technology that has seen rapid advancements in both technology and application recently [9,10,11]. Although it is a pertinent aspect in deepening our understanding concerning distinct functionalities of cellular ensembles, in particular that of human cells, we here promote the concept of chip-in-an-organ, or implantable bioreactor technology, as just being a further step towards advancing knowledge of living systems [12]. For this purpose, we employed a reliable 3R-compliant test, the hen’s egg test chorioallantoic membrane (HET-CAM) assay. In due course of this attempt, we also wanted to increase the standards of this in vivo test by designing a suitable micro bioreactor (MBR) and cost-effective peripheral instrumentation, such as a micropump system for automatic medium change within the reactor, and a camera system inside the incubator for live imaging of the chicken embryo. Features of the HET-CAM bioassay are its simple accessibility, high sensitivity, resourcefulness, low cost, and above that, the fact that growing blood vessels are directly contacted. Moreover, quantitative results can be obtained applying analysis of macroscopic images of CAM development as well as evaluation of vessel character based on microscopic techniques [13,14]. Therefore, the HET-CAM assay is an effective monitoring tool for assessing in vivo biocompatibility [15] and observing complex biological interactions [13]. As such, it provides an easy approach for testing chemicals [16], responses to biomaterials [17,18], living cells [19], and xenografts [20]. To date a wide variety of different types of MBR systems exist with different levels of complexity and versatile applications [21]. Hence, the objectives of this work were to conceptualize a scheme for easing the design and the prototyping by means of AM of fluidic MBRs for their use in an in vivo environment.

## 2. Materials and Methods

### 2.1. Reactor Prototyping and Additive Manufacturing

The 3D-printing was performed with the aid of SLA printers (Phrozen Sonic Mini and Phrozen Sonic Mini 4K according to the manufacturer’s instructions; Phrozen Technology; Taiwan) and thermoplastic extrusion printers (Prusa i3MK3S, Prusa Research a.s., Prague, Czech Republic). The 3D objects were either designed with the open-source programs OpenSCAD (OpenSCAD.org) and FreeCAD (freecadweb.org) or with AutoCAD (Autodesk, USA). The 3D digital vector files were processed with the open-source software Chitubox (chitubox.com). The protocol for PDMS HET-CAM reactor prototyping can be found in Supplementary Material and Methods/Results 1.

### 2.2. Cytotoxicity and Cytocompatibility

To determine the cytotoxicity and cytocompatibility of the 3D-printed materials, two different analytical tools were used. The first measure taken was the performance of the colorimetric MTT assay which determines cytotoxicity. Therefore, complete medium was conditioned in the 3D printed vessels for 24 h and subsequently added to a 96-well plate containing adherent hFOB cells. After another 24 h, the analysis was performed; for detailed protocol see Supplementary Material and Methods/Results 2—Medium conditioning.

Secondly, passaging experiments were conducted in direct contact to the materials. In this setting, hFOB cells were first cultivated in standard tissue culture dishes (TC), followed by detachment and transfer into 3D-printed wells for further cultivation, followed by detachment from 3D-printed wells and transfer back into tissue culture dishes. The full protocol can be found in Supplementary Material and Methods/Results 2—Cellular attachment and proliferation. Briefly, cells were seeded at a density of 1 × 106 cells and cultivated in standard 10-cm tissue culture (TC) dishes until reaching confluency of ~80%. After passaging the cells for the first time, they were transferred into printed resin wells PEGDA (3 min and 6 min UV exposure), Rapid Clear (3 min UV), as well as Phrozen Black (3 min and 6 min UV) at a density of 3 × 105 cells, and subsequently incubated therein for up to 48 h. The cell culture supernatant was collected and transferred into 6-well plates after 24 h and 48 h of incubation. Attached cells residing in the wells were detached with Trypsin/EDTA (Second passage) and transferred into 6-well plates. Pictures were acquired directly after transfer (2–3 h), and at different time points during culture.

### 2.3. Hen’s Egg Test—Chorioallantoic Membrane Assay (HET-CAM)—Biocompatibility 

A hen’s egg test chorioallantoic membrane assay was done to investigate the biocompatibility of the 3D prints. Consequently, the 3D prints were placed on the CAM surface of a chicken embryo for 72 h. The full protocol can be found in Supplementary Material and Methods/Results 2—Hen’s egg test—chorioallantoic membrane assay (HET-CAM).

### 2.4. Micro Bioreactor—Periphery and Instrumentation

The MBR was manufactured with the aid of an SLA printer and PEGDA resin. The reactor consists of an inlet and an outlet which are connected to a central chamber via channels. The MBR is coupled to a pump system, which enables application of, e.g., factors or cell suspension respectively. 

## 3. Results

### 3.1. PDMS Micro Bioreactor for HET-CAM 

**Figure 1:**
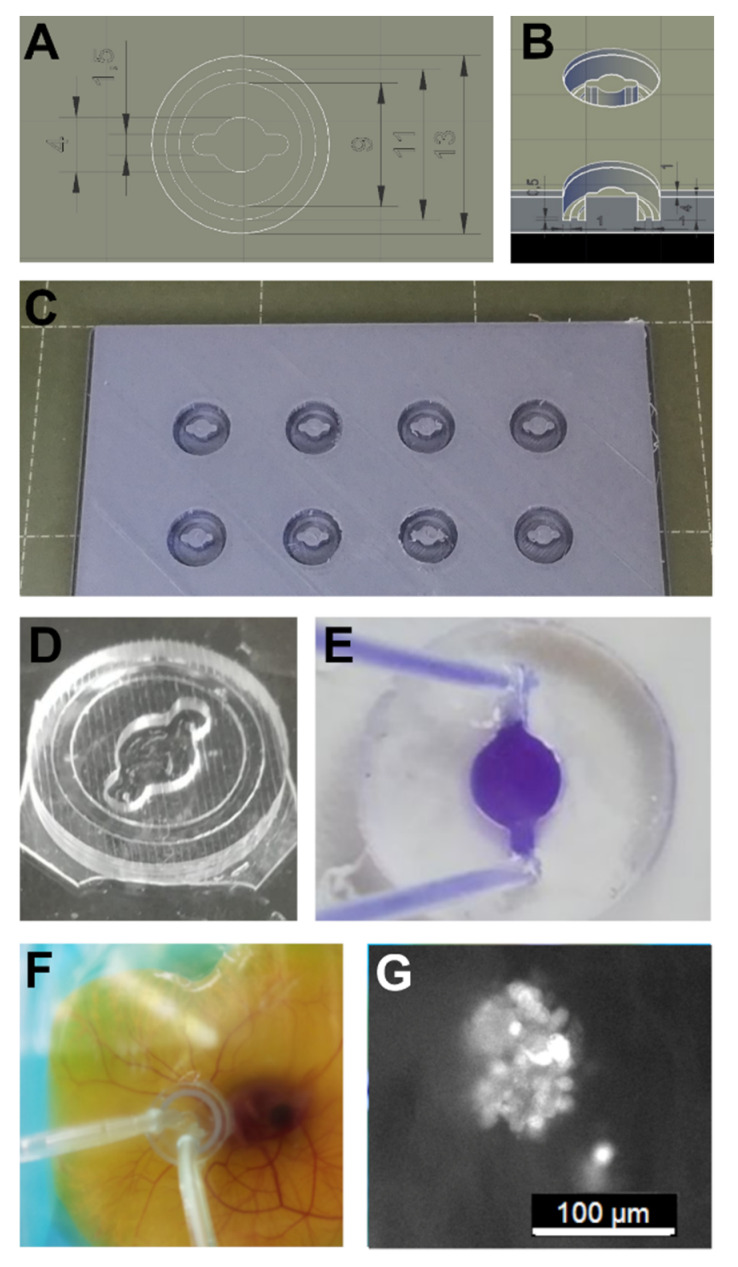
**Polydimethylsiloxane (PDMS) micro bioreactor (MBR) prototyping:** (**A**,**B**) The design was dome-shaped exhibiting a central flow chamber with two extensions for inlet and outlet connectivity. (**C**) An FDM mold was used for PDMS casting. (**D**,**E**) PDMS casts could be bonded leak-free with tissue adhesive to a plastic surface and connected to a pumping system via tubing. (**F**) PDMS MBR mounted onto an exo ovo cultivated HET-CAM could be operated without leakage for up to 2 days. (**G**) Human fetal osteoblasts expressing green fluorescent protein (hFOB:GFP) were injected into a PDMS chamber that had been planted onto a HET-CAM. After adherence to the CAM, cell clusters could be observed.

The technology of 3D-printing offers the advantage of easily generating objects with complex shapes and a network of channels. For proof of concept and feasibility, a material with validated biocompatibility was applied, polydimethylsiloxane (PDMS). This material is widely used for research purposes and is also used for many clinical applications [22]. For PDMS reactor production, molds were made by means of fused deposition molding (FDM) (Figure 1). To create a leak-free connection between the reactor and the CAM, different materials and methods were tested. N-butyl-cyanoacrylate super glue provided firm attachment of PDMS to the CAM without causing apparent interferences (Figure S1A). hFOB 1.19 cells expressing green fluorescent protein could be injected into the reactor and adhering cell clusters residing on the CAM could be observed (Figure 1G). For a detailed description see Supplementary Material and Methods/Results 1—PDMS HET-CAM Reactor Prototyping.

### 3.2. Cytocompatibility of 3D Prints

PDMS casting of complex geometries is greatly restricted, as nested or winding networks of channels or undercut shapes with skewed or slanted walls cannot be manufactured. Hence, rapid prototyping methods applying common AM technology were contemplated and resins amenable for 3D-printing had to be selected. In addition to commercial resins, Monocure Rapid 3D Resins (Clear or Black; RC or RB), Phrozen Black resin (PB), and eSUN water washable resin (eS), poly-(ethylene glycol)-diacrylate (PEGDA; P) has also been evaluated. These polyethylene glycol (PEG) derivatives have already been successfully used in a variety of tissue engineering and drug delivery-based applications. PEGDA, according to its higher polymeric chain length and thus molecular weight (MW), displays increasing grades of elasticity. All selected resins were stated to be odorless, emitting no volatile organic chemicals, displaying shrinkage below 0.5%, rapidly curing, and exhibiting high tensile strength. 

**Figure 2:**
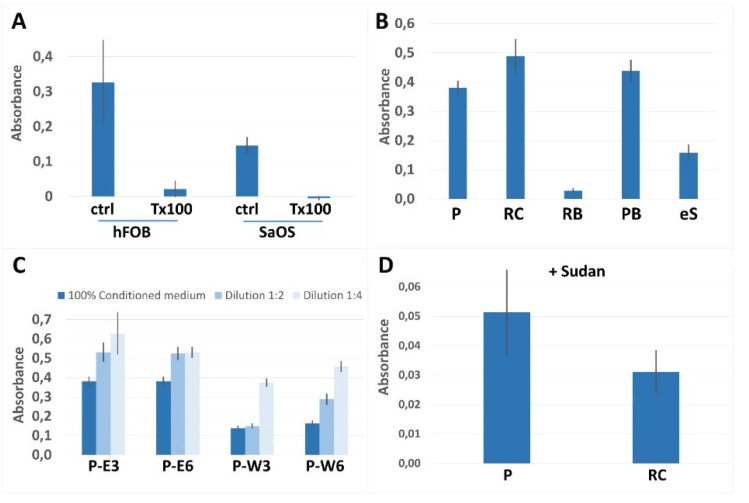
**Cytocompatibility:** (**A**) Metabolic activities were determined for hFOB 1.19 and SaOS-2 cells. Cells were treated for 24 h with growth medium, which had been conditioned with printed materials for 24 h. Controls were complete medium (ctrl), or medium containing 0.1% Triton-X-100 (T × 100). Conversion of MTT was assessed by measuring absorbance values at 492 nm. (**B**) Due to the broad dynamic range, cytocompatibility assessment was performed in hFOB cells applying media conditioned in wells PEGDA (P), Rapid Clear (RC), Rapid Black (RB), Phrozen Black (PB), and eSUN (eS). (**C**) For refining post-processing steps after 3D-printing, PEGDA wells were rinsed either with ethanol (E) or commercial resin wash (W) in combination with UV curing for indicated times in minutes. Media were diluted before treatment as indicated. (**D**) Addition of Sudan I to enhance manufacturing of the translucent resins PEGDA and Rapid Clear resulted in low vitality. Error bars indicate standard deviation; n = 6.

In the context of cytocompatibility testing, the cellular metabolic activity of human fetal osteoblast cell line 1.19 (hFOB) and the human osteosarcoma-derived cell line SaOS-2 were assessed and compared. hFOB cells appeared to be the more sensitive biosensor with an overall higher metabolic activity compared to SaOS-2 (Figure 2A). Working along these lines, post-print processing procedures for the individual printed materials could be accomplished (Figure 2B/Table S1). Since hFOB cells reacted particularly sensitively, dilutions of conditioned media were also evaluated (Figure 2C and Figure S2A).

RC resin showed positive results after sonication in ethanol in combination with UV curing for 3 min. Comparable results were achieved when sonicating in resin wash and UV curing for 6 min, yet polymerized RB resin appeared toxic regardless of extensive washing procedures. eS resin had been selected because it could be printed at high resolution and because it is water washable. However, all attempts failed to render the printed polymer suitable for application in cell culture. In stark contrast, cells incubated in media conditioned with PB exhibited enhanced metabolic activities compared to cells cultured in complete medium. Simply washing with ethanol and curing with UV light for 6 min resulted in high vitality. Treating hFOB cells with undiluted conditioned media derived from PB yielded 120% vitality compared to normal complete medium. We therefore further optimized the post-printing procedure for PB yielding best results by first sonicating in deionized water (MilliQ) for 1 h and a subsequent sonication in ethanol for 1 h (Figure 2B). Applying these test conditions for measuring the content of toxic compounds in post washing solutions, optimized washing procedures regarding times and solvents for the individual materials could be acquired (Figure 2C and Figure S2B and Table S1).

We were able to show that rinsing PEGDA with ethanol instead of commercially available resin wash solution resulted in better cellular vitality (Figure 2C). Finally, positive results were obtained for PEGDA and RC, as well as for PB, which prompted us to continue further evaluating these three materials. In contrast to PB, PEGDA and RC are translucent and thus suboptimal for producing high precision printing results. To render these resins suitable for high resolution printing, the commonly used UV absorber Sudan I was mixed with the resins before printing. We were however unsuccessful in establishing a post-printing protocol, which yielded the material compatible for the use in cell culture. 

**Figure 3:**
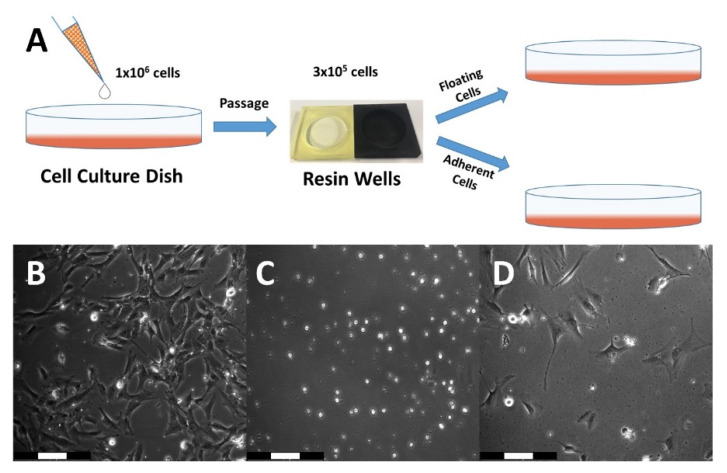
**HET-CAM material testing:** (**A**) Schematic representation of the workflow regarding ex ovo chicken embryo cultivation and material testing highlighting specific days of embryonic development (Day 0–10). (**B**) Full view of an embryo at EDD 9 with mounted PDMS rings for holding specimen in place; (**C**) 3D-printed PEGDA (black arrow) and (**D**) Phrozen Black flat rings were grafted to the CAM surface and cultivated for 72 h. Pictures were taken at embryonic day 10. In case of PEGDA, no apparent signs of adverse reactions or hemorrhage were detectable during the entire observation period, whereas Phrozen Black entailed severe defects and hemorrhage, as well as lysis endpoints.

To further investigate cellular vitality, adherence, and proliferation, the different resins were used to manufacture cell culture vessels with a diameter of 35 mm and a height of 6 mm, in which hFOB 1.19 cells were cultivated for passaging experiments (Figure 3A).

Comparing all three resins, the benchmark resin PEGDA showed by far the best results throughout the entire series of experiments (Figure 3B). PEGDA exhibited highest cellular vitality considering the number of viable cells in both cell fractions, either floating in the supernatant, or adherent residing in the printed culture vessels. Notably such a result could be exclusively observed for PEGDA. Cells derived from wells made of RC (Figure 3C) showed no apparent survival in both cell fractions. PB displayed only very low compatibility for cell cultivation as only a few cells remained viable (Figure 3D). Furthermore, after trypsinization of the remaining cells that resided in the wells, a significantly higher number of surviving cells was found in PEGDA wells compared to the other tested resins. At day 9 of cultivation after passaging, cells from resin wells made of PEGDA which had been UV-cured for 3 min reached 100% confluency. This result motivated us to continue with an in vivo analysis of PEGDA and PB resin to possibly endorse a potentially high degree of biocompatibility for these materials. 

### 3.3. Material Testing on HET-CAM

**Figure 4:**
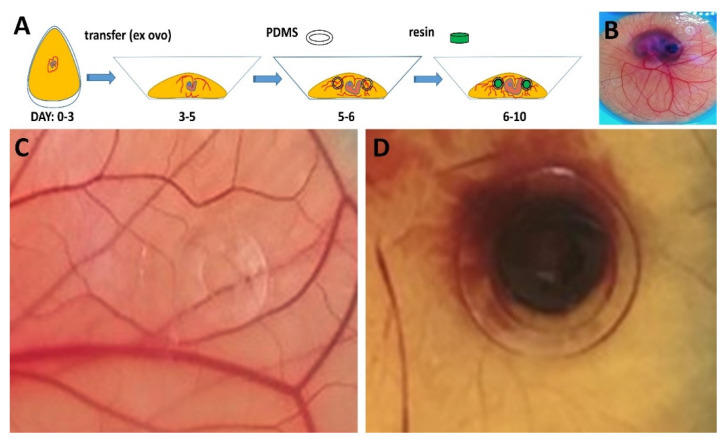
**Cell cultivation in 3D-printed vessels.** (**A**) Schematic representation of the workflow for this experiment. (**B**) PEGDA UV 3 min at day 2 after transfer from the resin well to 6 well plate. Cells from this well reached confluency at day 9 after transfer. (**C**) Rapid Clear UV 3 min at day 2 after transfer. No surviving cells were visible. (**D**) Phrozen Black UV 3 min at day 3 after transfer. The number of viable cells after transfer is much lower compared to PEGDA. Proliferation potential seemed to be inhibited.

After having assessed the cytotoxicity of the 3D-printed materials in vitro, putative effects after implantation into a living system, which is made to vastly grow, were determined. In this study, we intended to take advantage of testing the developing embryos fast growing vascular network. This developmental phase is highly sensible as the CAM grows and newly formed vessels sprout by means of endothelial cell proliferation. Due to these facts, the HET-CAM assay was performed ex ovo, in sterilized (70% EtOH; multiple hours of UV light) weighing dishes covered with provided lids or glass (Figure 4A). Pre-treatment of the xenografts included sterilization with 70% ethanol, subsequent washing with PBS, and incubation in complete medium. On embryonic development day 6 (EDD6), sterilized PDMS rings were placed over major vessels of the CAM surface to keep implanted specimen in place. On EDD7, 3D-printed xenografts were fit into the rings. For PEGDA, a test series of two biological replicates consisting of 4 technical replicates (n = 8) was conducted. After 72 h of incubation (Figure 4B), the experiments were terminated, both embryos showed no visible signs of disruption (Figure 4C). Based on all gathered results and observations, we consider PEGDA to be a biocompatible resin for biomedical applications or tissue engineering experiments. Within 72 h of CAM surface treatment with PB resin rings, the embryo had died. The vascular network collapsed completely, and clear signs of hemorrhage were observed (Figure 4D). Compatibility testing with respect to HET-CAM assays has been conducted beyond EDD10 by many research groups to date. At this developmental stage, the embryo evolves first consensual movements, approximately between EDD6 and EDD7, and first sensory neurons are formed from EDD8. Due to these facts, all experimental analyses in this study were terminated at EDD10, as the embryos begin to sense pain [23].

### 3.4. PEGDA Micro-Bioreactor for HET CAM and Cell Biological Applications

**Figure 5:**
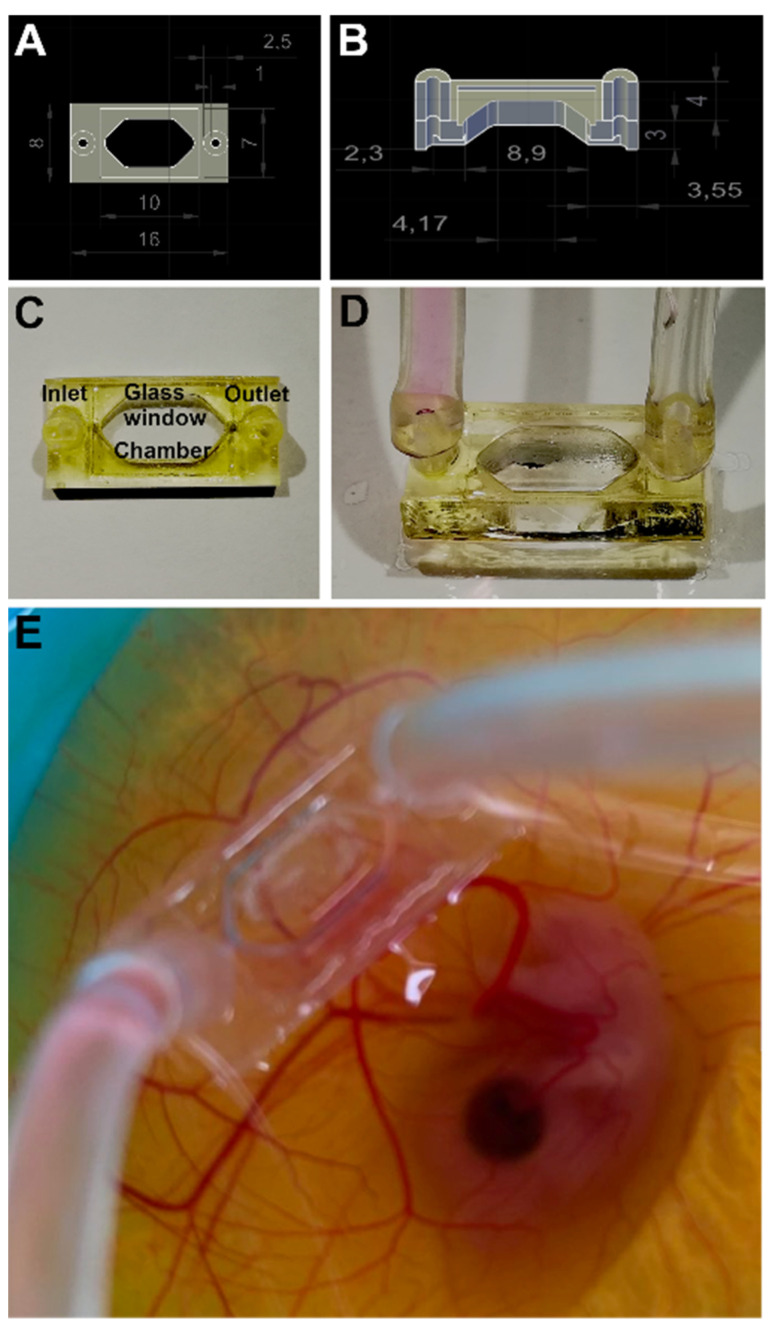
**PEGDA micro bioreactor prototyping and HET-CAM application:** (**A**,**B**) 3D CAD designs are depicted together with size marks. (**C**,**D**) The 3D prints exhibit inlet and outlet tubing connectors for media supply, and atop a glass ceiling. (**E**) A PEGDA MBR with already mounted tubing system was grafted on a CAM at embryonic day 7.

Next, a MBR for use in an in vivo environment to be produced from PEGDA-250 resin was redesigned (Figure 5). We sought to create a windowed perfusion MBR to ease observation of events taking place within the chamber once applied onto the CAM. A coverslip was laser-cut into the appropriate size and glued in as a glass ceiling of the MBR using a small amount of PEGDA resin, applied to the rims of the glass, and subsequently cured in a UV chamber. The perfusion MBR enables different application settings e.g., (i) cell seeding into the reactor chamber and monitoring of the interaction, (ii) flushing the chamber with different biogenic factors, (iii) application of gradients of reactants (low to high concentrations), (iv) testing materials incorporated into the chamber, or (v) vascularization of 3D cell culture (spheroids). The design and dimensions of the PEGDA MBR is depicted in Figure 5A,B. Post-print processing was done according to the presented protocol (Table S1). Thereafter, the glass window was fit on top of the chamber and MBRs were conditioned as described before. The MBR was tested on different substrates, i.e., tissue culture dishes and test dummies made of alginate-gelatin hydrogel, before applying on the CAM. The MBR was conjugated to the CAM of a chicken embryo at EDD6 with tissue adhesive (Surgibond) and stably remained there for at least 24 h. 

## 4. Discussion

Polymer extrusion is probably the simplest 3D-printing technique. It begins with a simple plastic filament that is heat-molten in a printing head and dispensed through a nozzle that reconstructs the desired object in a delineated fashion. Stereolithographic printers have significant advantages in particular regarding printing resolution, efficiency, and working conditions [24]. They most often comprise of a UV light source with a display and a printing platform. Energy-rich UV light activates polymerization of a liquid resin, which in due course turns the resin into a rigid polymer. When first passing light through a liquid crystal display that depicts a high resolution black-and-white-image, polymerization only takes place behind the translucent bright pixels. Hence, 3D objects can be built layer-by-layer through high resolution spatial photo-polymerization. As the rigid print body adheres to the printing platform, objects that exhibit petite features can also be rapidly manufactured [25].

The first MBR 3D-prototyping experiments which were previously conducted by our research team, conceptualized for proof of concept, utilizing FDM printing and PDMS casting were successful, suggesting moving towards SLA printing to enable a more detailed manufacturing of fluidic devices. High resolution printing provides several advantages for biomedical device fabrication, thus is limited by several factors such as the lack of biocompatibility.

We therefore firstly concentrated on introducing a novel workflow to meet reasonable biocompatibility standards, starting with post-print processing of 3D prints, followed by the assessment of cellular vitality in vitro, and eventually concluding the investigation with in vivo tests. Hence, this selective evaluation method enables determining adverse effects (i) exerted by toxic compounds, which leach out from the material, (ii) triggered also by surface properties when provided as a substrate for cell adherence and growth, and (iii) together when brought into a complex environment of a developing biological system. This analytical series exceeds the common standard by introducing further measures regarding cytocompatibility and in vivo compatibility. Only together these analyses provide a reliable first indication whether the material can actually be considered biocompatible. Indeed, bioreactor technology has already been introduced and applied to assess cytocompatibility and various aspects of biocompatibility [26]. Currently, 3D-printing technology is rapidly advancing in the field of biomedicine, in particular being adopted for tissue engineering approaches [27], including technical refinements which enable the production of very small-sized bioreactors [28] or inclusion of microstructures [29].

As most resin formulations and compositions are proprietary, biocompatibility must be independently checked after printing and crosslinking of the monomeric compounds together with a sufficient post-print processing of the polymeric structure. As outlined above, it is imperative to also include in vivo analyses [30]. As a first step, in particular to safeguard the integrity of cells and tissue, we set out to optimize washing and post-print-processing steps by applying various cleansing solvents and incubation times together with further post-curing UV exposures. Post-print processing was validated by means of the ISO 10993-5:2009 standard assay on two different cell lines hFOB 1.19 and SaOS-2. We chose hFOB cells because they have been derived from fetal tissue and it could be shown, by us and others, that they represent a multipotent surrogate in vitro model for stromal mesenchymal stem cells [31,32]. We were able to show that the hFOB1.19 cells more sensitively indicate cytotoxicity than SaOS-2 cancer cells. 

Biocompatibility was further assessed on a living organism in vivo with the aid of HET-CAM assays. Biocompatibility is defined as the ability of a material to perform its desired functions with respect to a medical therapy, to induce an appropriate host response in a specific application and to interact with living systems without having any risk of injury, toxicity, or rejection by the immune system and undesirable or inappropriate local or systemic effects [33]. This bioassay is very sensitive as the measures of the irritation score is given in time and hemorrhage, lysis, or coagulation can already be observed after a few minutes of material application. In this context, a wide variety of material formulations, such as collagen, gelatin sponges, various hydrogels, discs made of synthetic materials as well as drug-delivery methods in combination with small molecules, growth factors, or biosimilars, could be evaluated [34]. Furthermore, application of cells, most often derived from tumors as well as transgenic cells derived thereof, has been undertaken to study alteration of vascular growth and pattern [35]. Combining growth factors with cells in synthetic or natural scaffold materials is at the focus of tissue engineering, hence the CAM assay has also been adopted for this type of innovative research [36]. Work along similar lines of in vivo evaluation substantiated the previous findings from cell culture experiments. PEGDA was tolerated by the embryo during the full period of 72 h, with no visible signs of adverse effects. PB resin triggered adverse effects during a 48 h-incubation by leading to clear signs of hemorrhage and lysis. A superficial evaluation of the latter resin would have otherwise yielded a potentially positive result and release for application in biological research.

We expect that device prototyping and production by means of additive manufacturing will greatly promote PEGDA as a material applied in bioanalysis and regenerative medicine. Covalent binding of polyethylene glycol to a substrate is known as PEGylation [37]. PEGylation of nanoparticles applied in cancer treatment resulted in longer plasma half-life. It likely circumvents early clearing through activation of the complement system. Moreover, plasma clearance works through mitigating the opsonization of compounds [38]. It could however be shown that PEG-based hydrogels are degraded under oxidative conditions. This takes place both in vitro and in vivo [39]. It should be further noted that PEG-coatings elicited an anti-PEG based immune response [38]. After all, determining the grade of biocompatibility is an issue of primarily assessing incompatibility. That said, the question remains to be answered whether implanted material is actively segregated from surrounding tissue driven by a chronified foreign body reaction [40]. Taken together, to the best of our knowledge, this is the first report showing that 3D-printed MBR technology can be applied and used on the CAM of a chicken embryo. We are currently using the PEGDA MBR for test with human dermal microvascular endothelial cell line (HMEC-1) to investigate induced neovascularization and incorporation of HMEC-1 into the CAM vascular network to generate a novel vascularized organ-on-chip platform. 

## 5. Conclusions

We herein introduced a selective evaluation procedure for the assessment of biomaterial properties that are displayed after photopolymerization and washing of various 3D-printable resins. The interconnected method provides means for grading cytocompatibility before performing elaborate in vivo biocompatibility evaluations. Commercially available resins, for which neither formulation nor composition have been disclosed, could be evaluated in reference to a well-characterized material in the field, PEGDA. The step-by-step procedure facilitates certification of polymers with respect to their applicability in the context of specific analytical methods in basic cell biology and applied biomedicine. It could be shown that prints made of PEGDA are likely exhibiting a high degree of biocompatibility as this material showed no obvious signs of adverse reactions in a living and growing embryological setting. The method could also track down the suitability of additives, which, when mixed into resins, enhanced printing performance but had a pronounced impact on cells even after polymerization and extensive cleansing of the otherwise well-tolerated biomaterial. The MBR system comprises both an inlet for cell seeding and distribution of media or factors as well as an outlet for removing waste by means of controlled micropumps. The reactor has a glass ceiling for better visualization and monitoring within the chamber. Together with compatibility assessment, the prototypic design of novel in vivo MBR technology now enables complex organoid experiments, which can address a growing vascular system or propagate 3D-cell biology approaches taken into a microfluidic environment for the control of extrinsic parameters.

## Data Availability

Not applicable.

## Supplementary Materials

The following supporting information can be downloaded at: https://www.mdpi.com/article/10.3390/mi13081230/s1, Figure S1: Experimental setup to test of the in-vivo reactor on HET-CAM. (A) The reactor was prepared by using a pipette tip to distribute the adhesive on the bottom surface of the reactor. (B) The reactor was lowered onto the CAM. (C) After a connection had been established, the reactor was flushed with cell suspensions. After flushing the reactor with the periphery device labeled with (D) (red arrow), the embryo was placed back into the incubator. (E) Camera system for un-interrupted monitoring of the MBR in ex ovo HET-CAM setting. (F,G) Images taken immediately after start of incubation and 24 h later.; Figure S2: Cellular vitality index examined in hFOB cells after 24 h incubation with conditioned media derived from corresponding resin vessels used in this study (PEGDA, Rapid Clear, Rapid Black, Phrozen Black, and eSUN). Controls were complete medium (CTR GM) and a toxic control (CTR TX) containing detergent (complete medium + 0.1% Triton-X). The value of the control con-taining complete medium was set to 100% and the percentage values of conditioned media de-rived from the printed wells were calculated in reference to controls. Results exerting oxidoreduc-tase activities less than 70% are considered potentially toxic. (A) Various post-print processes were tested for each resin to achieve the best possible vitality for the cells. Illustrated is an example for PEGDA. (B) Based on vigorous testing, an optimal condition was determined for each resin. De-picted are the results of MTT analysis of optimized post-print processing methods for each resin used. (C) Optimized post-print processes for translucent resins PEGDA and Rapid Clear with Su-dan I. Despite different post-print processing, it was not possible to render resins mixed with Su-dan I cytocompatible; Figure S3: (A) Bartels mp-Multiboard micropump setup. With the mp-Multiboard 4 micropumps, a flow sensor, a pressure sensor, a thermal conductivity sensor, and two valves can be operated in parallel. In this experiment, only one pump was used. Liquid such as cell culture medium can be drawn and pumped into the reactor chamber, exchanging depleted medium with fresh medium at the same time. (B) The frequency and voltage can be set in the software. The voltage can be set individually for each pump. The frequency applies to all pumps. (C) With the integrated timer mode, pumps can be switched on and off at defined time points. (D) The reactor can also be sup-plied with medium using a self-made syringe pump. With the self-programmed Pure-Data soft-ware, the syringe pump can be controlled very precisely. Another advantage is that only a few components are needed and the pump costs significantly less than 100 €. (E) Pure-Data software: With this self-written program, the parameters can be defined very precisely; Figure S4: Web-based camera system setup inside the incubator, running on a Raspberry Pi. The motor (M) is mounted on the acrylic glass plate with the rotor facing down. One end of the rotat-ing arm (RA) is connected with the rotor and the other end with the Raspberry Pi camera (C). The motor can be programmed to halter the rotating arm at defined positions at defined time points for the camera to take macrographs; Figure S5: Dashboard of the experimental camera setup. When setting up the automation for an experiment, the rotation of the camera can be set to four different positions (0, 90, 180, 270) in correlation to degrees in a circle. A time interval between each macrographic acquisition can be freely selected; Figure S6: Website for HET-CAM observation. Settings are shown on the left side of the screen. HET-CAM: The positions can be moved manually by clicking on the cursor symbols. With the camera symbol, pictures can be taken. The green button is used to start the imaging automation; Figure S7: Time series imaging of a developing chicken embryo and chorioallantoic membrane, taken by the web-based camera system; Table S1: Optimized post-print processing for each resin used in this study. The reference [41,42,43,44,45] are cited in the supplementary materials.
