# Supplementary material for: A Poly-(ethylene glycol)-diacrylate 3D-Printed Micro-Bioreactor for Direct Cell Biological Implant-Testing on the Developing Chicken Chorioallantois Membrane
Source: Micromachines (Basel). 2022 Jul 31;13(8):1230. doi: 10.3390/mi13081230 (PMC9414255; doi:10.3390/mi13081230)
Supplement: Supplementary file 1 [file micromachines-13-01230-s001.zip › micromachines-1817427-supplementary.pdf]

micromachines

Supplementary Material

Supplementary Material and Methods/Results 1

PDMS HET-CAM Reactor Prototyping

Molds were 3D-printed with polylactic acid and shortly before PDMS casting coated with Ease Release 200 (Mann Release Technologies, Macungie PA). PDMS was mixed by using 9 parts of Nusil MED-6015 Part A and 1 part Nusil MED-6015 Part B (Avantor, PA USA). In order to remove bubbles, which emerged during mixing, the PDMS was placed in a desiccator for degassing. Thereafter, the PDMS mixture was carefully filled into the individual depressions. To accelerate curing of PDMS, the casting mold was placed into an incubator at 80 °C. After approximately 90 min, the hardened elastomer was removed. Holes for insertion of inlet and outlet silicon tubes were punched into the PDMS slab with a 2 mm biopsy-punch tool.

In addition to PDMS, agarose was also tested but found to be too brittle. Attempts with pig-skin gelatin were initially successful, but the objects started to dehydrate after 24 h and exhibited vast shrinkage. As expected PDMS caused no harm to the CAM and the embryos. Due to the simple dome structure of the reactors and by punching inlet and outlet channels, molding, casting and removal of cured PDMS could be easily accomplished in one piece. The only downside to PDMS was that the surface is hydrophobic, which calls for post-manufacturing-modification, such as functional coatings, or plasma treatment.

The final reactors exhibited a circular shape and measured 13 mm in diameter with a height of 5 mm. The chamber was 4 mm in diameter and had a height of 4 mm. The bottom rim eventually sits on the CAM surface thus closing the reactor. The inlet and outlet were 2 mm in diameter and were punched with the aid of a 2-mm biopsy punch (Kai Medical, JPN) connecting the chamber. The reactors also had an additional circumventing groove on the bottom side for better holding the adhesive in place before application of the reactor to the CAM surface.

Bonding to the HET-CAM Surface

To create a lasting connection between the reactor and the CAM, tests were undertaken on glass, plastic, and a human amnion (data not shown). Stable attachment of the PDMS structure to those materials was tested using glue (2-ethyl / n-butyl cyanoacrylate), pork skin gelatin, agarose, PDMS, plasma activation, and plasma activation in combination with surface modification and vacuum. Agarose, pork skin gelatin and PDMS, as well as the plasma modifications, did not provide leak-free bonding. Superglue and vacuum showed good results. However, the vacuum was too harsh in the ex ovo HET-CAM cultures as it damaged the membrane and the embryo died in due course of this application. Moreover, 2-ethyl-cyanoacrylate substantially harmed the CAM, but N-butyl-cyanoacrylate [41] appeared to serve well for the purpose of firmly attaching PDMS to the CAM without causing apparent interferences (Figure S1).

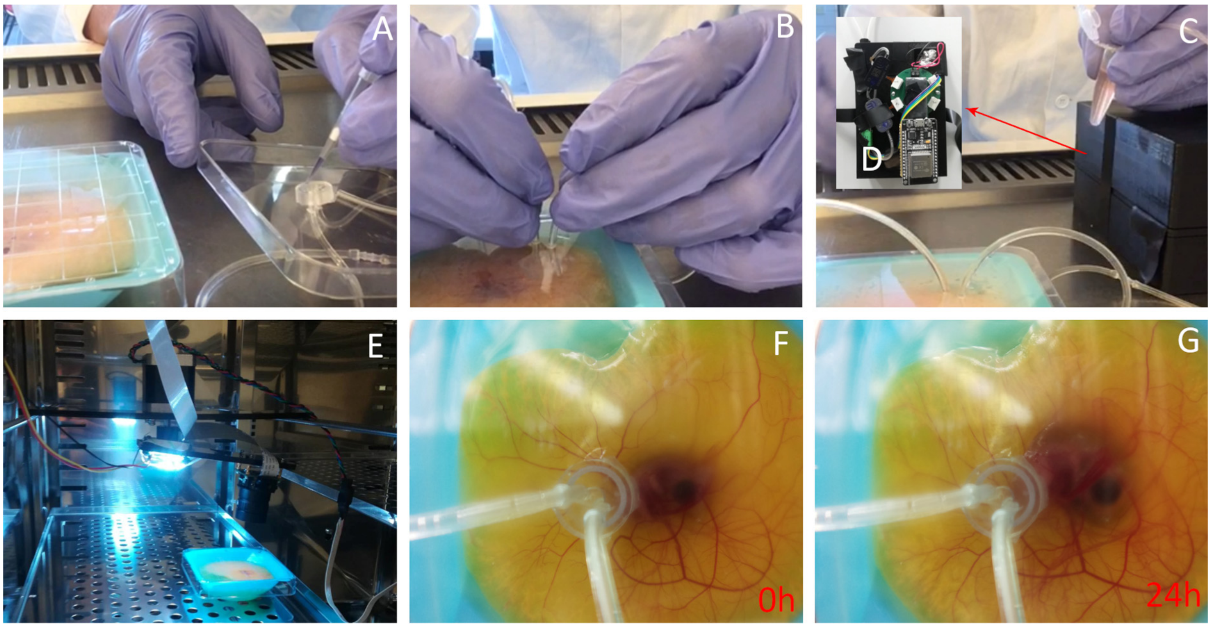

Figure S1. Experimental setup to test of the in-vivo reactor on HET-CAM. (**A**) The reactor was prepared by using a pipette tip to distribute the adhesive on the bottom surface of the reactor. (**B**) The reactor was lowered onto the CAM. (**C**) After a connection had been established, the reactor was flushed with cell suspensions. After flushing the reactor with the periphery device labeled with (**D**) (red arrow), the embryo was placed back into the incubator. (**E**) Camera system for uninterrupted monitoring of the MBR in ex ovo HET-CAM setting. (**F**,**G**) Images taken immediately after start of incubation and 24 h later.

Supplementary Material and Methods/Results 2

3D Printer Resins

PEGDA 250 was purchased from Sigma-Aldrich, Monocure3D (Australia) Rapid Clear, Rapid Black, Phrozen Black water washable resin, and eSUN resin were purchased from FEPshop.com. All resins were used as received. Additionally, the UV absorber Sudan I (Sigma-Aldrich, USA) was added to translucent resins PEGDA and RC.

PEGDA 250

Poly-(ethylene glycol)-diacrylate (PEGDA) is a polyethylene glycol (PEG)-based material that can be used for a variety of tissue engineering and drug delivery-based applications. There are different types of PEGDA. The higher the molecular weight (MW) of the PEGDA, the higher is the flexibility of the material. In 2016, Urrios et al. showed that PEGDA resin could be made biocompatible with specific post-print processing techniques [42]. It is frequently used as a pre-polymer solution, which can be used in the formation of a cross-linked polymeric system [43]. For successful 3D printing and polymerization of PEGDA 250, it was mixed with a photo-initiator. Therefore, we used 1% phenylbis (2,4,6-trimethylbenzoyl) phosphine oxide 97% powder purchased from Sigma-Aldrich (Irgacure 819). PEGDA with Irgacure can also be used in combination with a UV absorber to print small structures.

UV absorber Sudan I

When adding a UV absorber to prints, the transparent materials will not allow transmission of UV light into deeper layers. Without this dye, the actual printing resolution becomes limited as in the case of transparent resins, light penetrates into deeper, previously built layers, thereby crosslinking uncured resins in otherwise open channels or chamber structures. Hence, Sudan I was tested for its usability as UV absorber, as it exhibits broad absorption spectrum [44].

Rapid Clear and Rapid Black (RC and RB)

Monocure 3D Rapid model resins can be used with any low powered DLP 3D printer with UV wavelengths between 225 nm and 420 nm [45].

Phrozen Black (Ph. Blk.)

Unlike most standard resins, Phrozen Rapid Black Resin can be washed with water. This resin is customized for 405 nm LCD printers. Printed designs are sturdy and non-brittle.

eSUN Water Washable Resin (eSUN)

The eSUN resin can be used with 3D printers with a wavelength between 395 and 410 nm. The printed designs are sturdy and non-brittle.

Silane A174

In order to enhance adhesion of resins mixed with Sudan I to the printing platform, glass slides were coated with 98% 3-(trimethoxysilyl)-propyl methacrylate, also called Silane A174 (Sigma-Aldrich).

Cell vitality evaluation of 3D printable materials

PEGDA 250, Rapid Clear, Rapid Black, Phrozen Black, and eSUN resin were analyzed regarding their cytotoxic potential. Here, potential volatile and resolvable compounds, which leach out of cured printing materials were tested in cell culture. The

translucent resins PEGDA 250 and Rapid Clear were also tested in combination with Sudan I, which is a UV absorber that would enhance the effective printing resolution. As a biological sensor, human fetal osteoblast cell line 1.19 (hFOB) was chosen and applied. For cross-examination of the cellular sensitivity of hFOB cells towards materials used in this study, cell vitality experiments with prints made from PEGDA or Rapid Clear and PEGDA or Rapid Clear mixed with Sudan I were also conducted with SAOS cells, which is a cell line derived from primary osteosarcoma.

Medium conditioning

For the conditioning with medium, incubation vessels, consisting of 8 wells, each well with a diameter of 7 mm and a height of 6 mm, were printed. The wells were filled with 250 µL complete medium (DMEM: F12 growth medium, 10% fetal bovine serum (FBS), 1% Penicillin/Streptomycin (P/S), 1% L-Glutamine (L-GLU)) and incubated for 24 h at 37 °C. hFOB cells were plated in a 96-well plate overnight at 34°C, 20% O_2, and 5% CO_2 at a quantity of 8 x 10^3 cells per well. In due course, 100 µL of conditioned media derived from incubation vessels were added to the cells and incubated for 24 h. Potential cytotoxicity was analyzed using a standardized assay (Promega, CellTiter 96® AQueous One Solution Cell Proliferation Assay). Vitality measures are reflected by the cellular NAD(P)H-dependent mitochondrial oxidoreductase activity, converting the tetrazolium dye into formazan. The quantity of formazan product is directly proportional to the number of living cells in culture.

Controls were complete medium (CTR GM) and a toxic control containing detergent (complete medium + 0.1% Triton-X100). The conditioned media was added in concentrations: undiluted, a 1:2 and 1:4 dilution; the diluent being complete medium, respectively. The next day, 20 µL of Promega CellTiter 96® AQ_{ueous} One Solution was added to each well, including blanks and controls and incubated at 37 °C, 20% O_2, and 5% CO_2 for 3 h. After incubation, the absorbance was measured at 490 nm in a 96-well plate reader (Tecan).

Each resin was analyzed for its impact on cellular vitality of hFOB cells in culture, after incubating the cells with conditioned media derived from each corresponding resin vessel. Different post-print processes with different washing solutions and different UV curing times were tested to reach the highest cell vitality. Post-print processing washing solutions were analyzed in a standardized cytotoxicity assay also applying dilutions (1:2 and 1:4) thereof (Figure S2A). After gaining knowledge on the properties of the different resins, a standard post-print processing protocol was specified, and optimized conditions were determined for each resin separately (Figure S2B). This schema was also performed for PEGDA 250 and Rapid Clear mixed with Sudan I (Figure S2C).

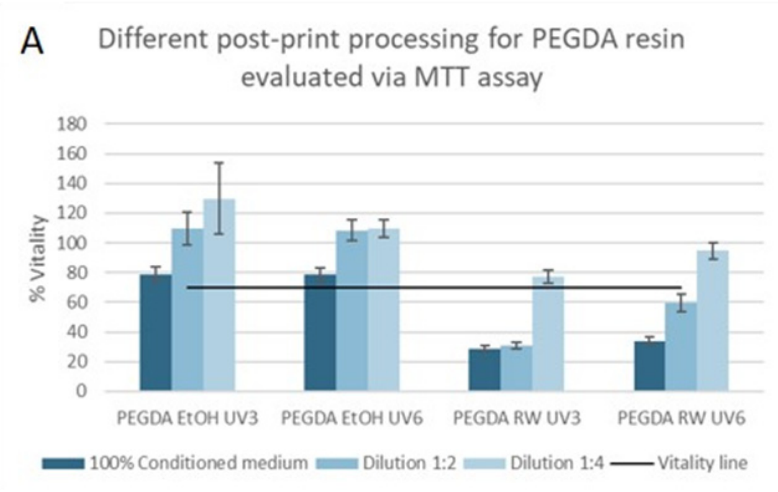

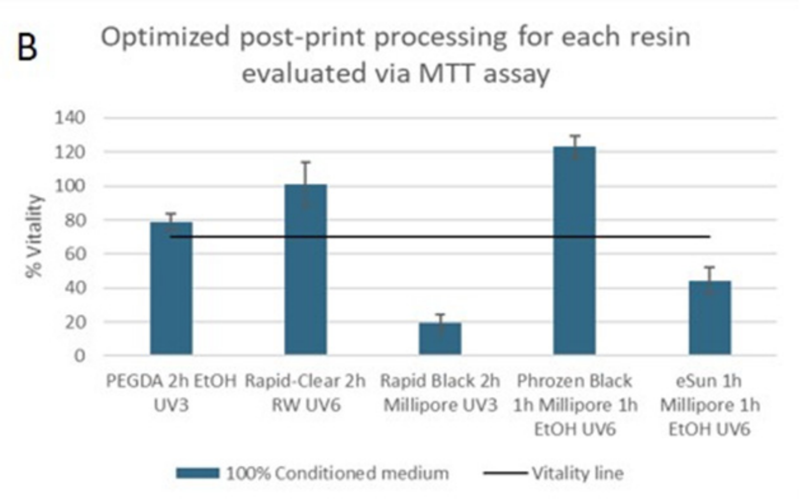

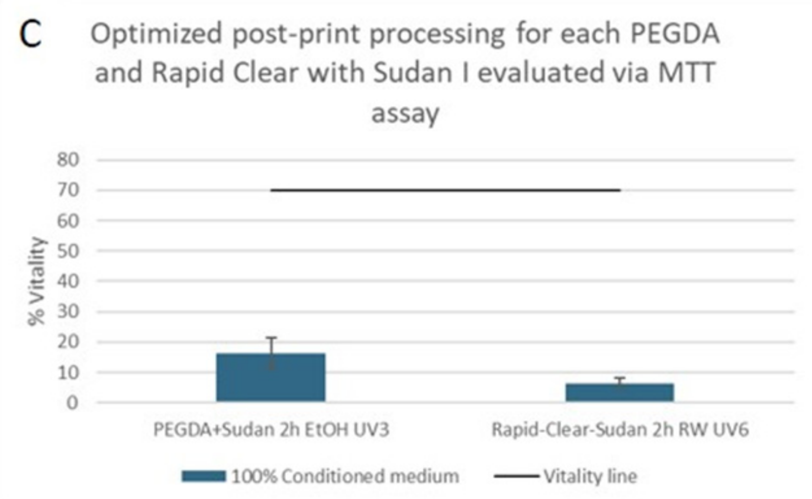

Figure S2. Cellular vitality index examined in hFOB cells after 24 h incubation with conditioned media derived from corresponding resin vessels used in this study (PEGDA, Rapid Clear, Rapid Black, Phrozen Black, and eSUN). Controls were complete medium (CTR GM) and a toxic control (CTR TX) containing detergent (complete medium + 0.1% Triton-X). The value of the control containing complete medium was set to 100% and the percentage values of conditioned media derived from the printed wells were calculated in reference to controls. Results exerting oxidoreductase activities less than 70% are considered potentially toxic. (**A**) Various post-print processes were tested for each resin to achieve the best possible vitality for the cells. Illustrated is an example for PEGDA. (**B**) Based on vigorous testing, an optimal condition was determined for each resin. Depicted are the results of MTT analysis of optimized post-print processing methods for each resin used. (**C**) Optimized post-print processes for translucent resins PEGDA and Rapid Clear with Sudan I. Despite different post-print processing, it was not possible to render resins mixed with Sudan I cytocompatible.

Post-print processing cleansing protocol:

Sonication in 70% Ethanol or MilliQ water for 1–2 h, rinsing with MilliQ water and subsequent curing with UV light for 3–6 min 100% (50 mW/cm²). Variations in duration of sonication and UV curing were dependent on the resin used.

PEGDA is a 3D printable analogue to PEG, which is known for its biocompatibility in many applications. Different washing solutions were used in order to establish a standard post-print processing method throughout this study for all the resins used. Interestingly, washing PEGDA prints with ethanol in comparison with a solution made especially for this purpose (Resin Wash; RW), resulted in better cellular vitality. The addition of

Sudan I to the resin resulted in significantly greater toxic potential compared to solely using the resin (Figure S2C).

Monocure Rapid Clear resin showed promising results for washing with ethanol and UV curing for 3 min as well as for washing with RW and UV curing for 6 min. All RC combinations with Sudan I resulted in extreme toxic values determined with MTT analysis (lower vitality values than toxic control), as well as the results obtained for Monocure's Rapid Black resin being very toxic. Therefore, these resin combinations were excluded from further investigations.

Phrozen Black resin results show a broad range of % vitality for the different post-print processing methods. Solely washing with ethanol and curing with UV light for 6 min resulted in vitality of over 120%, for the treatment with undiluted conditioned media (Figure S2C). Due to this broad range of results, consecutive testing of Phrozen Black resin for optimal post-print processing revealed a higher vitality for cells cultured in conditioned media coming from resin wells washed with ethanol than from the wells washed with water, as recommended by the manufacturer's site. The best results were yielded after washing the resin first in Millipore water for 1 h and subsequently cleansing in ethanol for 1 h. Results for this method exceeded any other resulting values from other resins.

eSUN resin was analyzed, because of its properties being very hard, having a high resolution in printing, and it being water washable. MTT analysis resulted in very low % vitality for all conditions and washing methods; therefore, investigations on this resin were not continued.

Table S1. Optimized post-print processing for each resin used in this study.

RESIN	PEGDA	PEGDA + Sudan I	Rapid-Clear	Rapid-Clear + Sudan I	Rapid Black	Phrozen Black	eSun
**SOLUTION**	EtOH	EtOH	resin wash	resin wash	Millipore water	Millipore/EtOH	EtOH
**TIME**	1 h	1 h	1 h	1 h	1 h	1 h/ 1 h	2 h
**UV EXPOSURE**	3 min	3 min	6 min	6 min	3 min	6 min	6 min

All in all, we obtained decent results for PEGDA and Rapid Clear, as well as for Phrozen Black, after optimizing post-print processing, as evaluated by means of the above-described cytotoxicity assays. These results suggested further investigation of cellular behavior when in direct contact to the resin.

Hen's egg test – chorioallantoic membrane assay (HET-CAM)

Fertilized hen's eggs were purchased at a nearby farm (Schlagerbauer Biohof, Salzburg, Austria) and cleaned and wiped with 70% ethanol before placing them in an automatically rotating incubator set to 37 °C and 55% relative humidity (% rH) for three days (Heka brut control; HEKA-Brutgeräte, Rietberg Germany). For ex ovo cultures, 10 x 10-cm weighing dishes were wiped with 70% ethanol and sterilized under UV light for 1–2 h prior to use. The rotation was haltered 30 min to 1 h prior to opening on embryonic development day 3 (EDD3) and again wiped with 70% ethanol. An egg cracker was used to carefully break through the shell in the entire circumference of the blunt side of the egg. Thereafter for another hour, the eggs were placed on a rack with the blunt side facing down to ensure the embryo is correctly oriented, positioned on top of the yolk after opening. The eggs were then transferred into a laminar flow hood, where after opening of the shell, the embryos were carefully dropped into the weighing dishes, and checked for proper heartbeat, integrity of the yolk sac, and initial vessel formation. The dishes were covered with sterile lids and transferred into an incubator set to 37 °C and 90% rH (Heraeus Instruments Cytoperm 2, Thermo Fisher Scientific, Waltham, MA).

Until EDD6, this procedure is the same, despite the modality of CAM treatment. For liquids and small xenografts, on EDD6, silicone rings made of PDMS (Sylgard 184 Silicone Elastomer; VWR International, Vienna Austria) were placed over major vessels to keep later treatment in place. For larger objects, the silicone rings were not needed and xenografts were placed directly on the CAM surface on EDD7.

In this study, PDMS casts and resin prints were investigated. On EDD7, a test series was conducted for each of the two resins. Four resin xenografts each were placed on the CAM surface, each into the previously applied PDMS rings. The larger PDMS casts and PEGDA MBRs were bonded to the CAM surface utilizing a n-butyl-cyano-acrylate-based tissue adhesive, Surgibond (SMI AG, St Vith, Belgium). After 72 h of incubation, all the experiments were terminated before the embryos could develop any sensation of pain.

Supplementary Material and Methods/Results 3

Micropump station

Flow rate and pressure were monitored with a Sensirion sensor SLF3s-0600F and the ABP pressure sensor from Honeywell; flow was further controlled with an active valve from Takasago SMV.

In order to pump liquids such as cell culture medium into the reactor with a defined flow rate and to renew medium at defined time points, we used a micropump system (Figure S3A) (Bartels, https://www.bartels-mikrotechnik.de/en/micropumps/). The self-priming mp6 micropumps comprise of 2 piezo actuators. Connected via a multi-board to a PC, they are operated by a software provided by Bartels (Figure S3B,C) (https://www.bartels-mikrotechnik.de/downloads). The software can be used to change the amplitude and frequency of the piezo actuators, thus specifying the flow rate. The run time of the micropump can be switched on and off at defined time points or intervals. The fluidic system can also be operated by means of a low-cost self-built syringe pump (Figure S3D) and an open-source program (Figure S3E).

A

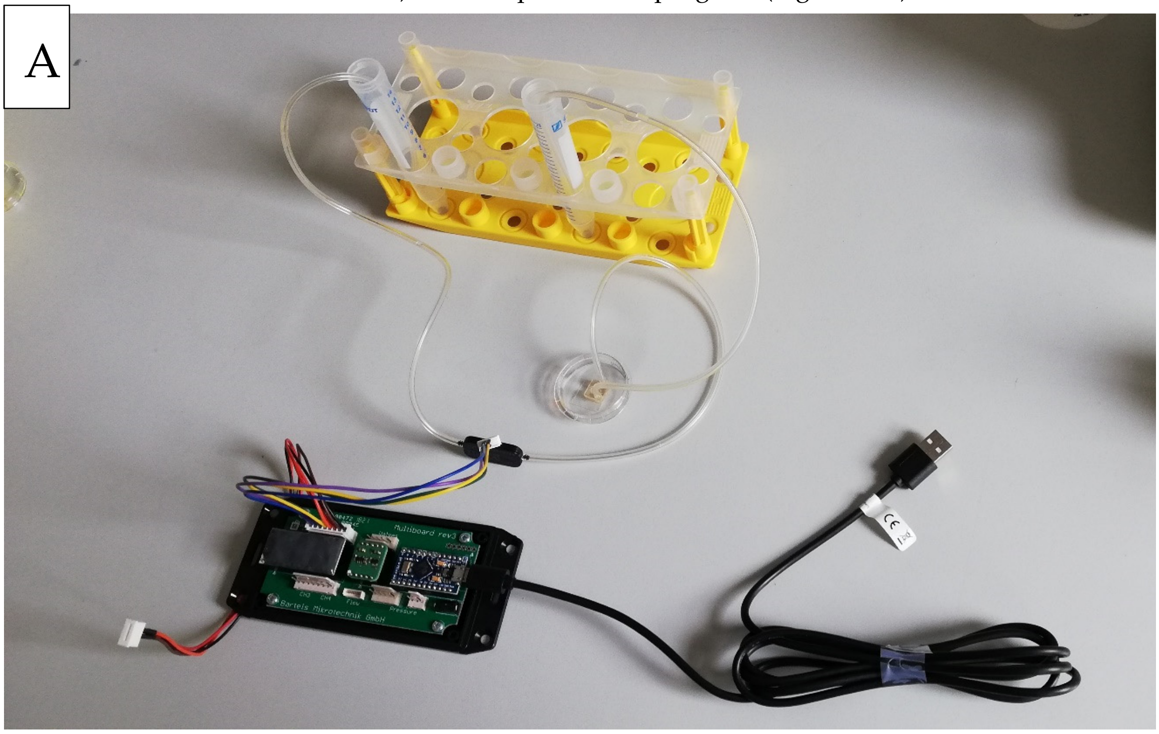

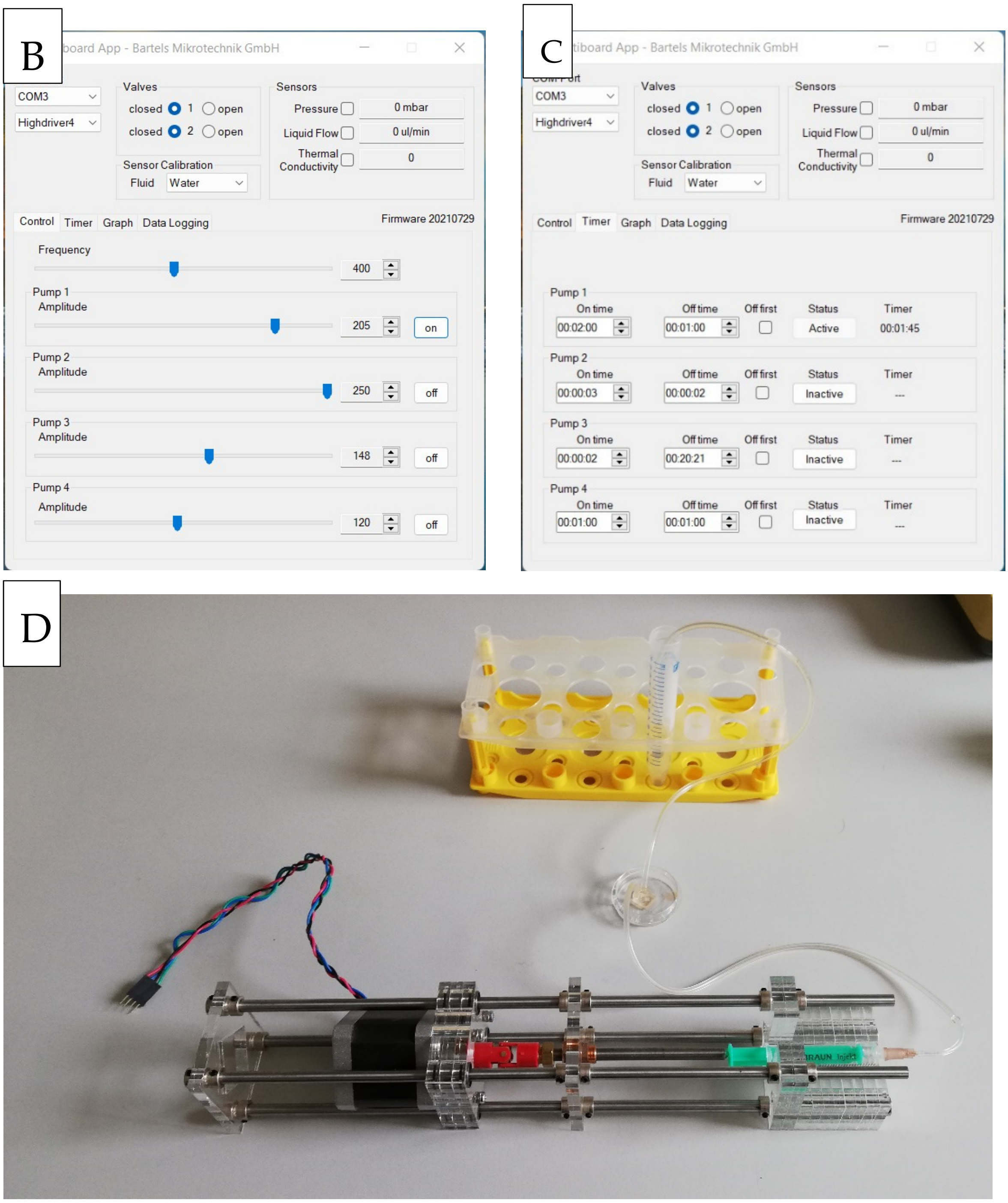
B
board App - Bartels Mikrotechnik GmbH
COM3
Highdriver4
Valves
closed 1 open
closed 2 open
Sensor Calibration
Fluid Water
Sensors
Pressure 0 mbar
Liquid Flow 0 ul/min
Thermal Conductivity 0
Control Timer Graph Data Logging
Firmware 20210729
Frequency 400
Pump 1 Amplitude 205 on
Pump 2 Amplitude 250 off
Pump 3 Amplitude 148 off
Pump 4 Amplitude 120 off
C
tiboard App - Bartels Mikrotechnik GmbH
COM3
Highdriver4
Valves
closed 1 open
closed 2 open
Sensor Calibration
Fluid Water
Sensors
Pressure 0 mbar
Liquid Flow 0 ul/min
Thermal Conductivity 0
Control Timer Graph Data Logging
Firmware 20210729
Pump 1 On time 00:02:00 Off time 00:01:00 Off first Status Active Timer 00:01:45
Pump 2 On time 00:00:03 Off time 00:00:02 Off first Status Inactive Timer ---
Pump 3 On time 00:00:02 Off time 00:20:21 Off first Status Inactive Timer ---
Pump 4 On time 00:01:00 Off time 00:01:00 Off first Status Inactive Timer ---
D

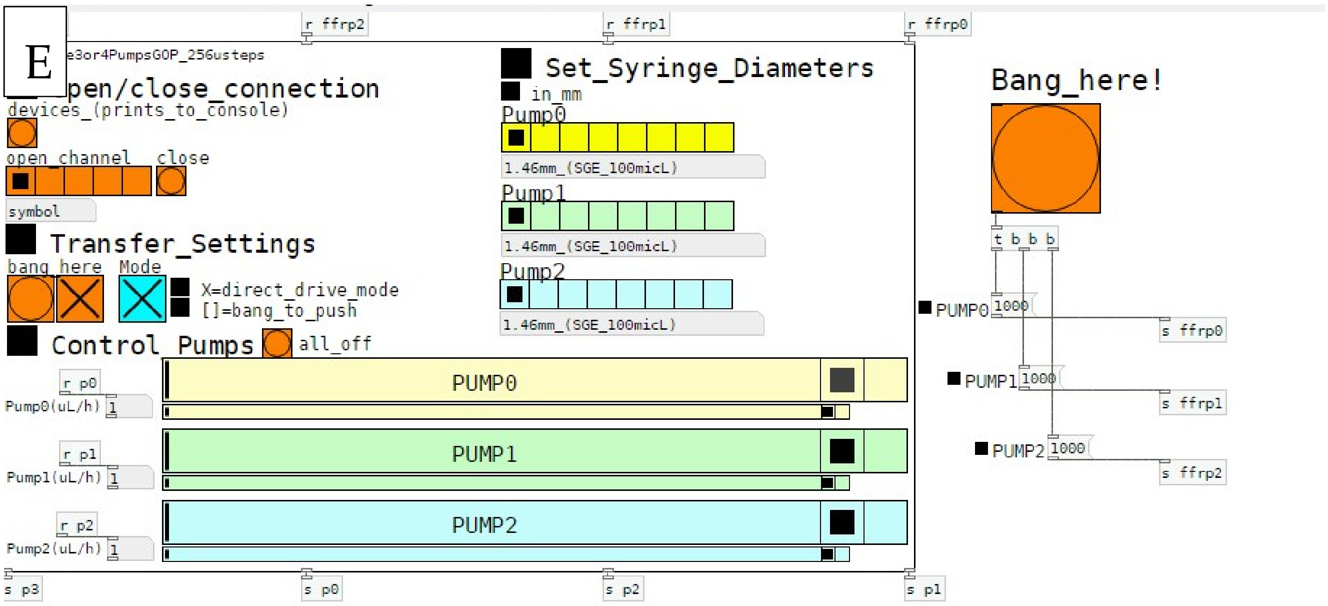

Figure S3. (**A**) Bartels mp-Multiboard micropump setup. With the mp-Multiboard 4 micropumps, a flow sensor, a pressure sensor, a thermal conductivity sensor, and two valves can be operated in parallel. In this experiment, only one pump was used. Liquid such as cell culture medium can be drawn and pumped into the reactor chamber, exchanging depleted medium with fresh medium at the same time. (**B**) The frequency and voltage can be set in the software. The voltage can be set individually for each pump. The frequency applies to all pumps. (**C**) With the integrated timer mode, pumps can be switched on and off at defined time points. (**D**) The reactor can also be supplied with medium using a self-made syringe pump. With the self-programmed Pure-Data software, the syringe pump can be controlled very precisely. Another advantage is that only a few components are needed and the pump costs significantly less than 100 €. (**E**) Pure-Data software: With this self-written program, the parameters can be defined very precisely.

Supplementary Material and Methods/Results 4

HET-CAM Imaging

The camera system consisted of a small single-board computer, a Raspberry Pi (Raspberry Pi foundation) in conjunction with a Raspberry Pi camera (Raspberry Pi foundation). Lights and light controls in the incubator comprised of an LED set behind an LEE filter to diffuse the light. The imaging apparatus consisted of a stepper motor, the Raspberry PI camera, a camera holder printed from FDM, and two acrylic glass plates. One acrylic glass plate was clamped to the guide rails of the incubator. The centerpiece of this plate held the stepper motor in place which was mounted with the rotor side down. The rotating arm is also cut from acrylic glass plates. One end of the arm was connected to the rotor, the other end of the arm was mounted to the camera holder with connected camera with the lens facing down (Figure S4). The stepper motor attached to the rotating arm is able to move to four different positions at individual time intervals. The stepper motor and the camera are controlled by the Raspberry PI. The Raspberry PI unit was connected to the network, thus allowing remote operating via the web browser and VPN access for the purpose of video imaging (Figure S4 and S5) allowing time-resolved macroscopic image grabbing (Figure S6). Photos were taken every hour with the web-based camera system for a duration of 62 h. We created a time series imaging of the developing chicken embryo and the chorioallantois membrane (Figure S7).

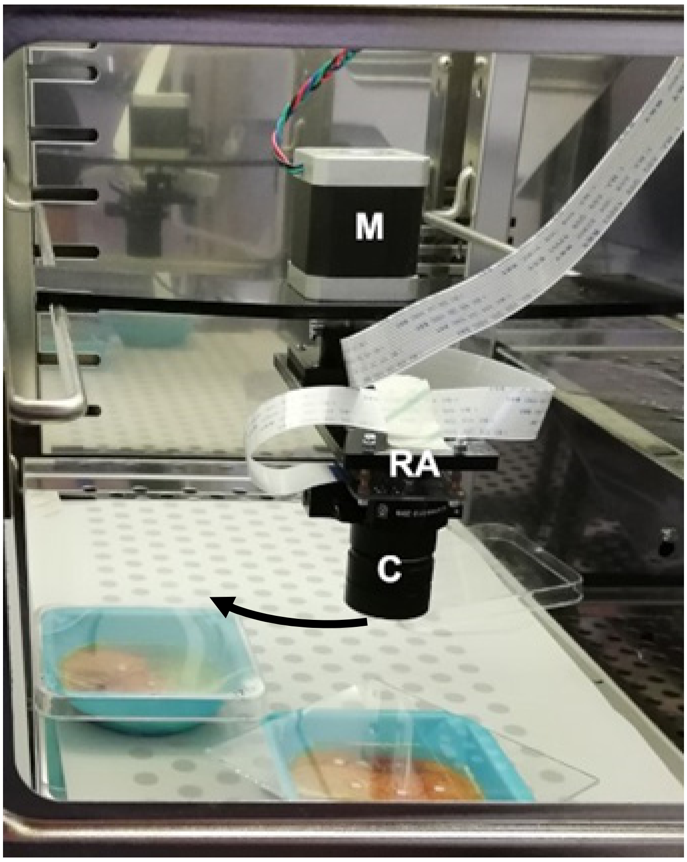

Figure S4. Web-based camera system setup inside the incubator, running on a Raspberry Pi. The motor (M) is mounted on the acrylic glass plate with the rotor facing down. One end of the rotating arm (RA) is connected with the rotor and the other end with the Raspberry Pi camera (C). The motor can be programmed to halter the rotating arm at defined positions at defined time points for the camera to take macrographs.

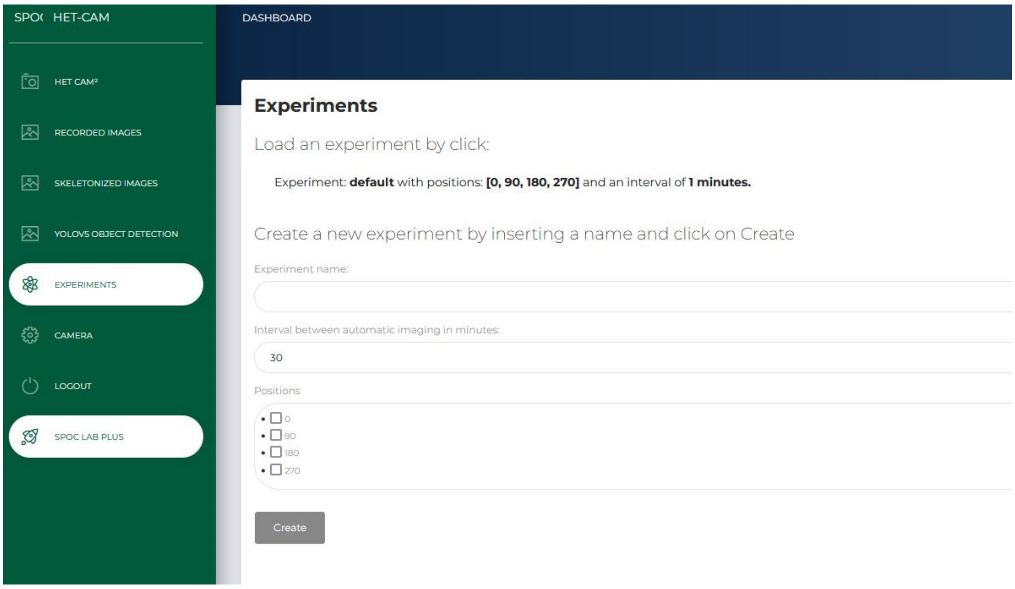

Figure S5. Dashboard of the experimental camera setup. When setting up the automation for an experiment, the rotation of the camera can be set to four different positions (0, 90, 180, 270) in correlation to degrees in a circle. A time interval between each macrographic acquisition can be freely selected.

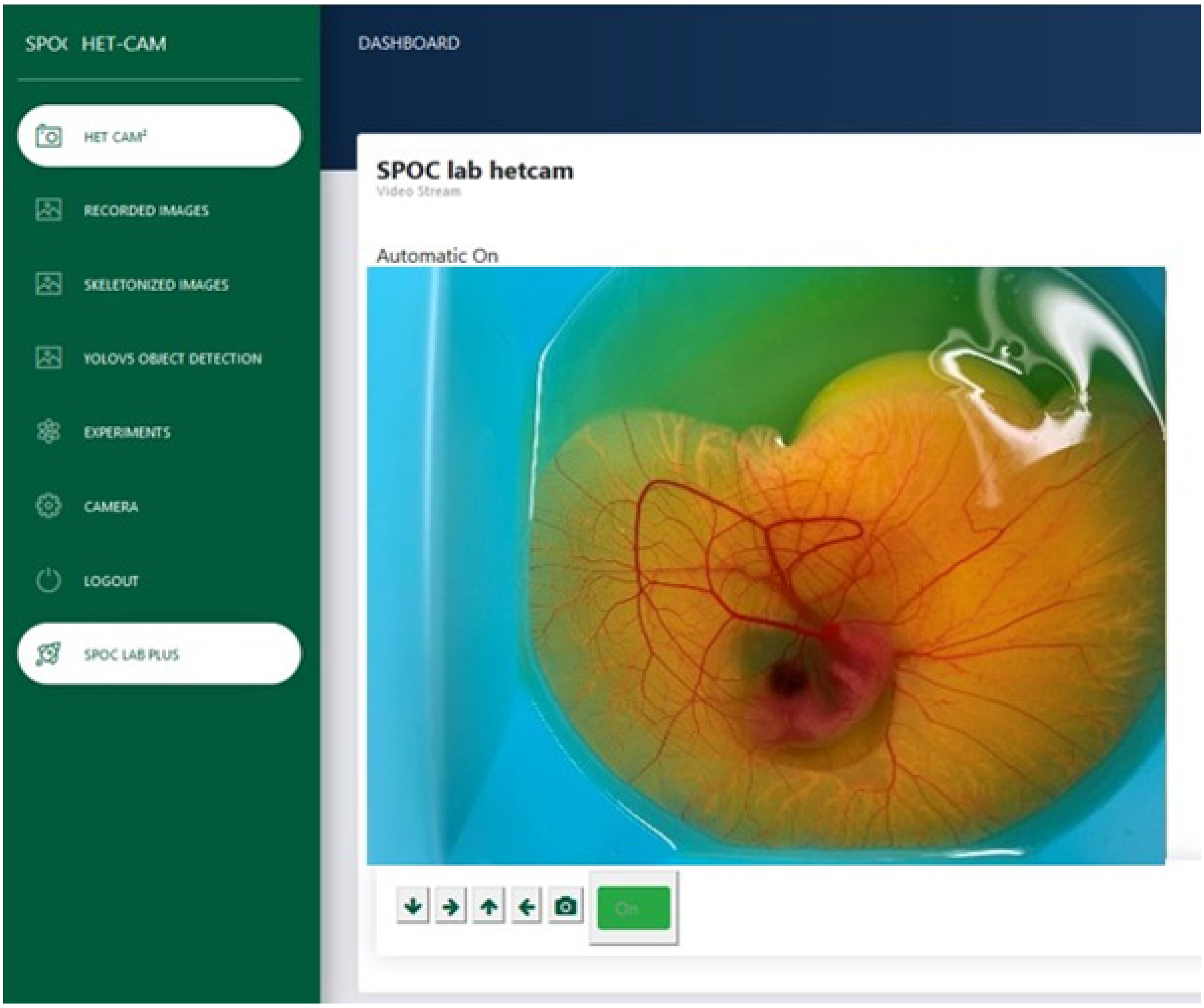

Figure S6. Website for HET-CAM observation. Settings are shown on the left side of the screen. **HET-CAM:** The positions can be moved manually by clicking on the cursor symbols. With the camera symbol, pictures can be taken. The green button is used to start the imaging automation.

- **RECORDED IMAGES:** Photos already taken can be viewed here.
- **SKELETONIZED IMAGES:** The branching of the vascular system can be mapped.
- **VOLOV'S OBJECT DETECTION:** Living objects can be detected.
- **EXPERIMENTS:** Recording positions and times can be defined.
- **CAMERA:** Camera settings can be defined.

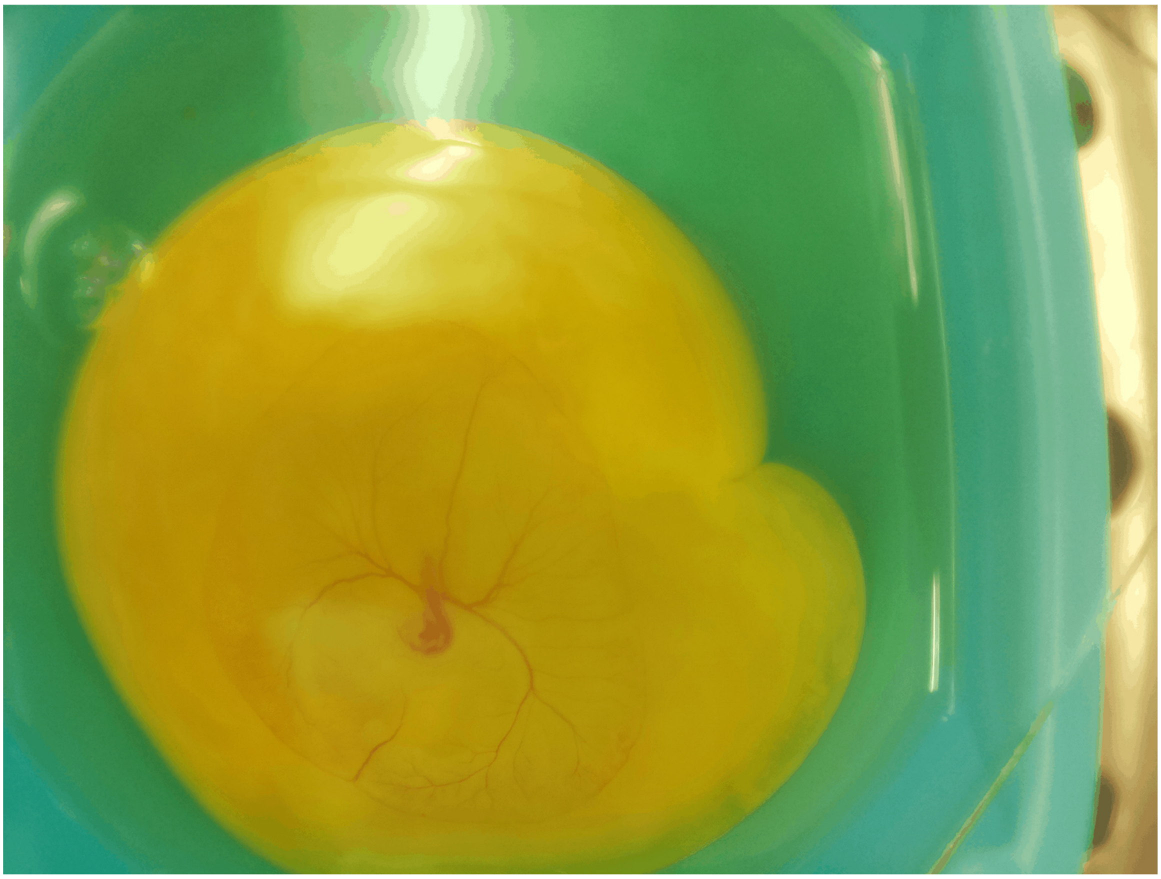

Figure S7. Time series imaging of a developing chicken embryo and chorioallantoic membrane, taken by the web-based camera system.
